# Edge roughness analysis in nanoscale for single-molecule localization microscopy images

**DOI:** 10.1515/nanoph-2023-0709

**Published:** 2024-01-04

**Authors:** Uidon Jeong, Ga-eun Go, Dokyung Jeong, Dongmin Lee, Min Jeong Kim, Minjae Kang, Namyoon Kim, Jaehwang Jung, Wookrae Kim, Myungjun Lee, Doory Kim

**Affiliations:** Department of Chemistry, Hanyang University, Seoul 04763, Republic of Korea; MI Equipment R&D Team, Mechatronics Research, Samsung Electronics Co., Ltd., Hwaseong 18848, Republic of Korea; Research Institute for Convergence of Basic Sciences, Institute of Nano Science and Technology, and Research Institute for Natural Sciences, Hanyang University, Seoul 04763, Republic of Korea

**Keywords:** single-molecule localization microscopy, semiconductor, cell membrane roughness, edge roughness analysis, line edge roughness, power spectral density

## Abstract

The recent advances in super-resolution fluorescence microscopy, including single-molecule localization microscopy (SMLM), has enabled the study of previously inaccessible details, such as the organization of proteins within cellular compartments and even nanostructures in nonbiological nanomaterials, such as the polymers and semiconductors. With such developments, the need for the development of various computational nanostructure analysis methods for SMLM images is also increasing; however, this has been limited to protein cluster analysis. In this study, we developed an edge structure analysis method for pointillistic SMLM images based on the line edge roughness and power spectral density analyses. By investigating the effect of point properties in SMLM images, such as the size, density, and localization precision on the roughness measurement, we successfully demonstrated this analysis method for experimental SMLM images of actual samples, including the semiconductor line patterns, cytoskeletal elements, and cell membranes. This systematic investigation of the effect of each localization rendering parameter on edge roughness measurement provides a range for the optimal rendering parameters that preserve the relevant nanoscale structure of interest. These new methods are expected to expand our understanding of the targets by providing valuable insights into edge nanoscale structures that have not been previously obtained quantitatively.

## 1 Introduction

The developments in super-resolution fluorescence microscopy (SRM) have allowed researchers to visualize cellular structures and molecular interactions at resolutions exceeding the diffraction limits of conventional light microscopy [1], [2], [3], [4]. This has enabled the study of previously inaccessible details, such as the organization of proteins within cellular compartments, spatial arrangement of molecules on cellular surfaces, and dynamics of cellular processes [5]. Recently, this technique has been successfully used for imaging nonbiological nanomaterials, including polymers and semiconductors [6], [7], [8], [9].

SRM can be broadly classified into two categories: the first group focuses on engineering the illumination patterns, and the second group relies on single-molecule localization methods, known as single-molecule localization microscopy (SMLM), which include stochastic optical reconstruction microscopy (STORM) [3] and photoactivated localization microscopy (PALM) [4]. Among them, the SMLM techniques achieve precise localization of individual fluorescent emitters by temporally separating their activation based on stochastic “on–off” fluorescence photoswitching, thereby preventing overlapping each other [10]. In the SMLM, the final super-resolution image is reconstructed from individually localized points with high precision, obtained through the detection and localization of single fluorescent molecules. The resulting high-resolution image exhibits a pointillistic nature owing to the collection of the precise localization of molecules, which is different from the conventional fluorescence or electron microscopic images [11]. Therefore, the structural analysis approach to the SMLM data must be different from the conventional structural analysis methods used for intensity grid-valued pixel-based images obtained from conventional microscopy. This necessitates the development of a new method to analyze the structural properties of the SMLM images [12]. For instance, various computational cluster analysis methods have been developed recently for SMLM images to identify protein clusters on a molecular scale and understand the protein-to-protein interactions [13], [14], [15], [16], [17], [18], [19]. Although the modern computational cluster analysis methods for pointillism data provide ultrastructural information regarding targets in terms of protein clustering or organization, most of them are limited to the spatial statistics of the size, quantity, and distribution of clusters.

Meanwhile, edge structural analysis still remains challenging for SMLM images thus far despite the significance of edge information in various areas, including biology and nanomaterials. For example, the line-edge roughness (LER) analysis, which refers to the variation or irregularity in the edge profile of patterned lines and features, is of paramount importance in semiconductor metrology because of its direct impact on device performance and yield [20]. In addition, cell membrane roughness analysis plays a critical role in understanding the cellular physiology, disease processes, cell–substrate interactions, biophysical properties, and applications in nanotechnology and drug delivery because it is considered an indicator of a cell’s health state [21]. Because of the importance of edge structural information, several image analysis methods for edge roughness have been developed; however, they are only for general intensity grid-valued pixel-based images, such as electron microscopy images, conventional fluorescence microscopy images, and atomic force microscopy (AFM) images [22], [23], [24], [25]. Because these conventional edge roughness analysis methods for intensity grid-valued pixel-based images cannot be applied to pointillistic SMLM images, it necessitates a distinct analysis method for edge roughness from SMLM image data.

The edge structure analysis method for pointillistic SMLM images can be approached using either the localization coordinates or localization-rendered images [12]. As the localization coordinate–based structure analysis, the conventional cluster analysis method can be applied for edge detection, such as SR-Tesseler [14], molecular density distribution, and edge trace functions (that is, boundary and smooth). However, these options are sensitive to the background localization signal as well as to the local localization density, making accurate edge detection difficult, particularly in low-localization-density regions (Figure S1). Owing to such limitations in edge detection, quantitative analysis of nanoscale edge roughness, such as LER, from SMLM images has been challenging with conventional localization coordinate–based structure analyses. Additionally, structure analysis based on localization coordinates tends to be time-consuming when it comes to analyzing regions with high localization density, primarily due to the extensive volume of coordinate information involved. Despite the increasing run-time of the coordinate-based method for the high localization number dataset, it is important to employ highly dense localization data to obtain reasonable structural analysis results. This is explained by the concept of Nyquist sampling, which states that at least twice the frequency must be sampled to accurately measure a certain frequency [26], [27], [28]. Such Nyquist criteria are more significant in edge structural analyses than in general cluster analyses. For the nanoscale edge roughness analysis, the edge should be identified at the nanoscale using localization with a significantly higher density than usual. If the localization density is not sufficiently high, there are many empty areas, resulting in incorrect edge identification. In contrast, in a localization-rendered image-based method, there is less empty area in the reconstructed image because of the rendered localization based on the photo-switching properties of a single molecule, such as the photon number, on-time length, and point spread function (PSF). Therefore, a localization-rendered image-based method for edge structure analysis can be often preferred to the localization coordinate–based structure analysis, particularly for datasets with limited localization density in pointillistic image data.

Therefore, we developed a new approach for nanoscale quantitative edge structure analysis using localization-rendered SMLM images in terms of the LER and power spectral density (PSD). We first developed an edge identification method for the SMLM image data and calculated the line-edge roughness using the identified edge structure. Then, we demonstrate this method for simulated SMLM data to investigate the effect of point properties, such as size, density, and localization precision on roughness measurement, providing the optimal rendering parameters that preserves the relevant nanoscale structure of interest. Using this correlation based on simulation data as calibration, we could successfully measure the roughness of true structures from experimental SMLM images of actual samples, including the semiconductor line patterns and cell membranes. We could further demonstrate this analysis method for detecting defects on a semiconductor wafer, as well as distinguishing the differences in cell membrane roughness depending on their locations with respect to the moving direction of cells. The proposed novel methods are expected to expand our understanding of a target by providing valuable insights into edge nanoscale structures that have not been previously obtained quantitatively.

## 2 Results and discussion

### 2.1 Effect of localization property in pointillism super-resolution images on roughness measurement

In a localization-rendered image-based method, edge identification is significantly affected by the properties of the localization image, including the localization precision, density, size, and background level. To understand these effects on edge roughness measurement, we simulated theoretical STORM images of line patterns based on various properties of the localization image.

**Figure 1:**
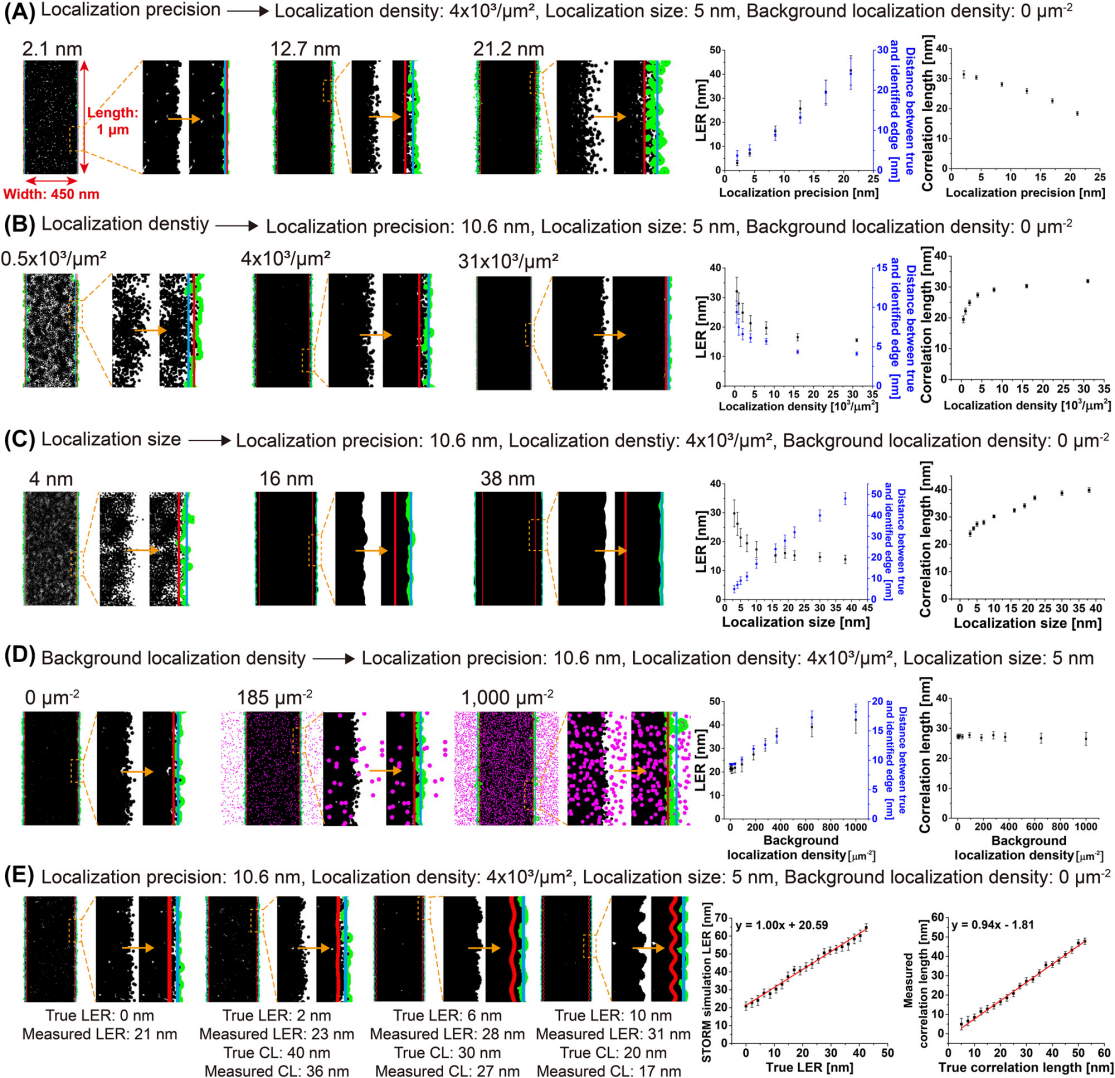
Effect of localization property in SMLM images on roughness measurement. (A–D) Simulated STORM images of a line pattern (width = 450 nm, length = 1 μm, LER = 0 nm for true structure) with (A) different localization precision, (B) localization density, (C) localization size, and (D) background level (true edge, identified edge, and fitted central line for each edge are shown in red, green, and blue, respectively; their calculated LER, correlation length [CL], and distance between the identified edge and true edge are shown on the right to examine the effect of localization precisions, localization density, and background level, on the LER, edge identification error, and correlation length). (E) Simulated STORM images of a line pattern with different theoretical LER values (0–20 nm) using experimental image parameters (localization precision = 10.6 nm, localization density = 4.0 × 10^3^/μm^2^, localization size = 5 nm in diameter, background localization level = 0 μm^−2^) (true edge, identified edge and fitted central line for each edge are shown in red, green, and blue, respectively; their measured LER values and correlation lengths are shown on the bottom to compare with the true values for each edge structure).

First, the edge roughness can be sensitively changed by the localization precision because the distribution of single-molecule localization generated from the localization uncertainty could result in additional roughness in the STORM image compared to the true sample roughness. Therefore, the additional roughness effect from localization uncertainty should be considered when estimating the true sample feature roughness from the STORM image. The localization precision of a single molecule is generally measured from a histogram of the standard deviation (*σ*) from the centroid positions, in which the FWHM of its Gaussian fitting is measured as the spatial resolution ( $\text{FWHM}=2\sqrt{2\ln2}\sigma \approx 2.355\sigma$ ), as previously reported [29]. Therefore, we generated simulated theoretical STORM images of line patterns with various localization precision values from 2.1 nm to 21.2 nm. For each simulated STORM image, the edges of the line patterns were detected by fitting the boundaries of the line edges using the Canny algorithm, followed by the calculation of the LER, which represents the deviation of the line edge from a straight line (Figure S2A). The LER was calculated as three times the standard deviation of the edge as previously reported [25]. As expected, the LER values measured from the simulated STORM images showed a dependency on the resolution of the STORM images, that is, the FWHM of the single-molecule distribution (Figure 1A). With the change of the localization precision of the single-molecule distribution from 2.1 to 21.2 nm, which is the measured localization uncertainty, the detected edge of the line patterns in the simulated STORM images using the Canny algorithm is far apart from the true edge, yielding increasing LER values from 3 to 42 nm for the zero roughness of the true sample line (Figure 1A). This implies that a localization precision of ∼8 nm in general STORM images could result in an additional roughness of ∼15 nm on an average.

Next, we investigated the effect of localization density on edge roughness measurements by generating simulated theoretical STORM images of line patterns with various localization densities. As stated earlier, a low localization density in pointillistic STORM images can be problematic in defining the correct edge structure owing to the empty area, resulting in a higher roughness value measurement than the true value. In the simulated STORM images of the line patterns with various localization densities, we observed such an error in identifying the edge boundary when the localization density was low (Figure 1B). Therefore, as the localization density decreases, the identified edge boundary diverges from the ideal central line, thereby increasing the LER values. However, we found that the measured LER value approaches the constant value when the localization density is higher than 4.0 × 10^3^/μm^2^, suggesting the localization density of 4.0 × 10^3^/μm^2^ could be enough to measure the LER accurately in the nanoscale.

As the next rendering parameter, we investigated the effect of the localization size on the edge roughness measurement by generating simulated theoretical STORM images of line patterns with various localization sizes. The localization size in the STORM images is usually selected to show a reasonable structure at a given image magnification. Although larger localization is expected to fill the empty area, the identified edge boundary from large localizations can be separated from the real edge boundary. We observed such effects from the LER measurement and identified the edge boundary from the simulated theoretical STORM images of line patterns with various localization sizes (Figure 1C). Significantly small-sized localization results in a high LER value owing to the larger empty area between the localizations, whereas a localization that is significantly large causes a low LER value owing to the smoothness effect but loses the real roughness information. Additionally, a large localization size can result in the identified edge boundary being far from the real edge boundary. Because a localization size larger than 5 nm in diameter does not significantly affect the LER value and a localization size smaller than 5 nm in diameter does not cause a significant error in the identification of the edge boundary from the true structure, the localization size of 5 nm appears to be appropriate for the nanoscale roughness analysis.

Finally, we explored the effect of background level on the edge roughness measurement by generating simulated theoretical STORM images of line patterns at various background localization levels. The background localization level can vary depending on the fluorophore labeling method from nonspecific binding, threshold for single-molecule localization analysis, and autofluorescence and contamination of samples. If the background localization level, in terms of the background localization density, is significantly high, it can disturb accurate edge boundary identification. This effect of a high background localization level on the LER measurement can be observed in our simulation data. As shown in Figure 1D, higher background localization could result in incorrect edge boundary identification, generating a higher roughness value. However, we found that the background localization density lower than 90/μm^2^ does not affect the roughness measurement significantly, whereas the background localization density higher than 90/μm^2^ results in additional roughness linearly (Figure 1D). Therefore, if the background localization density is known, we can estimate the additional roughness due to this background level; hence, the real roughness value can be calculated after calibration.

Collectively, the additional LER values from the localization property, including the localization precision, localization density, localization size, and background level in the STORM images, were systematically investigated. The true edge roughness can be estimated by considering this effect based on the calibration curve.

Although the LER is the most commonly used parameter to represent edge roughness features by providing roughness height information, there are other useful roughness features that have recently been used to improve the understanding of roughness effects, such as the power spectral density (PSD). Therefore, we analyzed the PSD of the edge boundary for pointillistic STORM images. They can be measured from the identified edge boundary using the Canny algorithm, in the same manner as in the LER measurement.

First, we performed a PSD analysis, which has recently been used to understand line roughness in the semiconductor industry on the simulated STORM images to characterize roughness as a function of frequency [30]. The PSD is defined as the variance of the edge or linewidth per unit frequency, and this frequency dependence of roughness is known to contain significantly more information than that in the LER alone [24]. For example, the correlation length obtained from a PSD profile can provide roughness information independent of white noise by determining the transition decay from lower to higher spatial frequencies, thereby reducing the noise bias from the LER measurements [24]. Increasing the correlation length implies a smoother (i.e., a less rough) edge appearance [24]. Based on the generated PSD curve for each STORM image, the correlation length can be measured from the curve fit, as previously described (Figure S2A) [24]. We performed the PSD analysis on the various simulated STORM images to investigate the effects of localization properties, such as the localization precision, localization density, localization size, and background level on the measured correlation length (Figure 1A–D). When generating the PSD curve, we attempted to analyze the STORM images with a small grid size and large domain size to minimize the statistical bias, as previously reported [23]. In general, we observed that the correlation length varied depending on the localization properties, inversely correlated to the LER value. For example, the correlation length tends to decrease, whereas the LER value increases as the localization uncertainty increases, implying a rougher edge appearance (Figure 1A). In a similar way, the correlation length was observed to increase, whereas the LER value decreases as the localization density increases, implying a smoother edge appearance (Figure 1B). Also, the correlation length tends to increase as the localization size increases as shown in Figure 1C, implying a smoother edge appearance. Such decreased roughness from the STORM images rendered with a larger localization size is consistent with the decreased LER value, probably owing to the smoothness with the loss of the real roughness information. Because such a change in correlation length is correlated with the localization properties, including localization precision, density, and size, the correlation length for the true structure can be estimated once the localization properties used for the images are known. Meanwhile, we found that the measured correlation length from a PSD curve was relatively independent of the background level, in contrast to the LER analysis (Figure 1D). This feature was similar to the noise-independent correlation length measured from the SEM images. Therefore, we found that the measured correlation length from the STORM images was unbiased by the background level in the STORM images unless it is too significantly high, which is an advantage of the PSD analysis over the LER analysis for the STORM images.

### 2.2 LER and PSD analysis for various types of line patterns

Using the simulated STORM images of a line pattern with zero roughness, we investigated the effect of the localization property of the SMLM images on the roughness measurement. Based on this result, we further applied our LER and PSD analysis methods to line patterns with various roughness values to generate a calibration curve for the relationship between the true and measured roughness. We first generated simulated STORM images of a line pattern with different LER values (0–42 nm) using our experimental image parameters (localization precision = 10.6 nm, localization density = 4.0 × 10^3^/μm^2^, localization diameter = 5 nm, and background localization level = 0/μm^2^). For each simulated STORM image, the edge boundary was identified, and the LER and PSD analyses were performed. As shown in Figure 1E, our roughness measurements could distinguish between the different nanoscale roughness values by generating an increased LER and decreased correlation length for the increased roughness of the true structures. Interestingly, the measured LER and correlation length changed linearly with the true roughness, allowing the true roughness to be estimated from our measurements. For example, if the LER and correlation length are measured as 25 and 30 nm, respectively, from the STORM images with known image parameters (localization precision, localization density, localization size, and background level), the true LER and correlation length are estimated as 4 and 34 nm, respectively, based on our calibration data. Therefore, we demonstrate that our roughness measurement from the STORM images can distinguish between the different nanoscale roughness values; the true roughness can be estimated from our analysis. Next, we investigated whether the LER and correlation length changed sensitively for the line patterns with various types of defects, such as the nanoparticles, bridges, or breaks. To test this hypothesis, we generated simulated STORM images of line patterns (roughness = 0 nm) with various types of defects and measured the LER and correlation length, as shown in Figure S3. Notably, our roughness analysis showed a distinct LER and correlation length for the line patterns with defects, which were different from those of the line patterns without defects. For example, the LER measured from the line patterns with defects (LER = 110 nm) exhibited a larger value than those without defects (LER = 21 nm), whereas the correlation length measured from the line patterns with defects (CL = 10 nm) exhibited a shorter value than those without defects (CL = 27 nm). Therefore, the existence of defects can be identified from the LER measurements and PSD analysis, demonstrating the capability of our method to inspect defects in semiconductors.

### 2.3 Edge roughness analysis for experimental STORM images of nanopatterns in semiconductor wafer

Using our new methods for the analysis of edge roughness in terms of the LER and correlation length, we first attempted to analyze the edge roughness of line patterns in the semiconductor wafers. STORM images of silicon and silica line patterns were obtained using a recently developed super-resolution fluorescence imaging method for nanopatterns in semiconductor materials [7], [8]. Briefly, the wafer was coated with positively charged poly-l-lysine (PLL) and then labeled with negatively charged BSA conjugated with AF647 dye for silica-specific labeling. For Si-specific labeling, the wafer was first coated with negatively charged Nafion and then labeled with positively charged bovine serum albumin (BSA) conjugated with AF647 dye. Using these silica- or silicon-specific dye-labeled wafers based on charge interactions, we obtained the STORM images of the silica or silicon nanopatterns.

**Figure 2:**
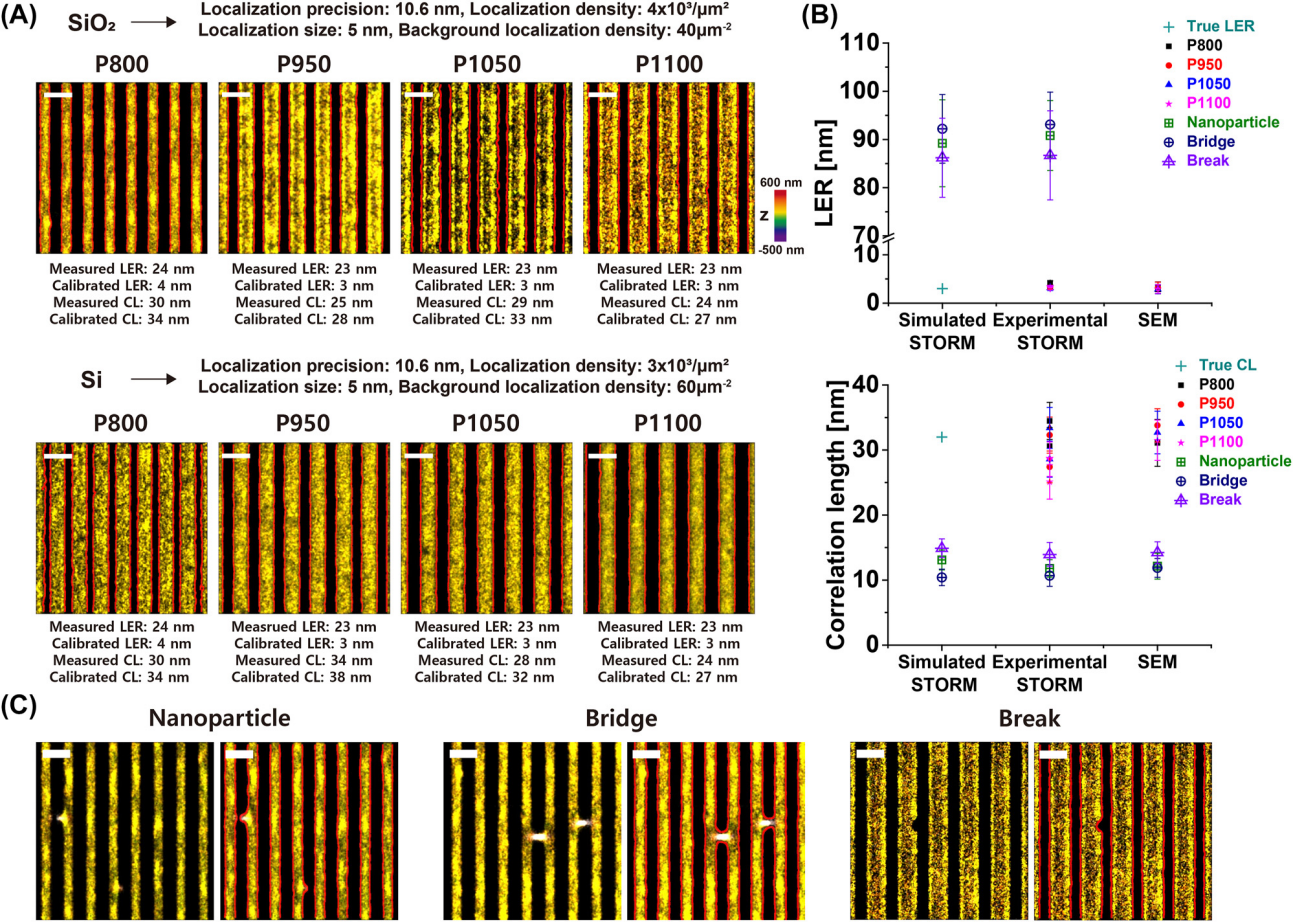
Roughness measurement from experimental STORM images of semiconductor nanopattern arrays. (A) Super-resolution STORM images of different sized nano-patterned arrays (pitch: 800 nm, 950 nm, 1050 nm, 1100 nm from left to right) specifically silica-(top) or silicon-(bottom) labeled with Alexa Fluor 647, red: identified edge (measured and calibrated LER and correlation length are shown together). (B) Comparison of the LER and correlation length values obtained from simulated and experimental STORM images and SEM images of different sized nano-patterned arrays on silicon wafers. For experimental STORM images, the calibrated LER and correlation length based on the used localization parameters are presented. The simulated STORM images were generated for the line structure with the calibrated LER value (3 nm) that was obtained from the experimental STORM image (empty shapes: calibrated LER and correlation length for a line pattern with defects, including nanoparticle, bridge, and break).(*n* = 15, mean ± SD) (C) Representative super-resolution STORM images (left) and overlay of STORM and identified edge images (right) of nano-patterned arrays with various defects, including nanoparticle, bridge, and break, specifically silica-labeled with Alexa Fluor 647. Scale bar: 1 μm.

Using these super-resolution images of the nanopatterns, we performed roughness measurements, including the LER and correlation length. It is important to measure the line roughness in semiconductor metrology because it is known to be a major contributor to the lithography error budget, resulting in current leakage and voltage fluctuations [31], [32], [33]. To estimate the roughness value for the true structure from the measurement with experimental STORM images, we first measured the FWHM of the Gaussian fitting to a histogram of the deviations from the centroid positions as the spatial resolution. Although we used a high-refractive-index imaging buffer to minimize spherical aberration, the measured FWHM was ∼25 nm, which is larger than the reported values for the sample on the cover glass, probably because this sample was at a distance from the coverslip [11]. By assuming the Gaussian distribution, the localization precision is obtained as 10.6 nm from FWHM of ∼25 nm. To generate the STORM images, we used a localization size of 5 nm in diameter and pixel size of 3 nm. The localization density in silica- and silicon-specific STORM images was 4.0 × 10^3^/μm^2^ and 3.0 × 10^3^/μm^2^, respectively. The background localization density in silica- and silicon-specific STORM images was measured as 40/μm^2^ and 60/μm^2^, respectively. Using these image parameters and the calibration data of the effects of the localization properties on each roughness value, we calibrated the LER and correlation length for the true structure from each measured value (Figure 2A).

To confirm that our roughness measurements provided accurate values, we also measured the roughness values from the SEM images of the same types of wafers. The SEM imaging was performed at a slow scan rate (0.026 frame/s) to minimize the noise compared to the sample roughness. We also prepared SEM images with a small grid size and large domain size to minimize the statistical bias, as previously reported [23]. We found that our measurement results of the LER and correlation length from the STORM images were consistent with those measured from the high-resolution SEM images, implying the reliability of the roughness analysis from the STORM images (Figure 2B). As previously reported, we also noted that the measured roughness values from the SEM images obtained at a fast scan rate and with a large pixel size (i.e., low magnification) showed large measurement errors owing to the image noise and absence of nanoscale structure information; thus, they were not consistent with the measurements from the STORM images (Figure S4). The accurate LER values could be obtained from the SEM images at a slower scan rate than 0.026 frame/s and with a pixel size smaller than 8 nm (Figure S4), implying that the SEM imaging time longer than 14.5 min is necessary to obtain accurate nanoscale LER value for the 1100 μm^2^ area of the wafer sample. In contrast, the STORM images of the same area of the wafer sample can be obtained within 6.6 min, and the nanoscale LER values can be obtained from these images with an accuracy as high as that obtained from the SEM images acquired over a significantly longer time (∼14.5 min). Therefore, our analysis method allows for accurate nanoscale LER and correlation length measurements from the STORM images obtained within a considerably shorter acquisition time than the SEM images.

Furthermore, we found that our roughness measurements exhibited distinct values for defects in the semiconductor wafers, demonstrating its potential as a nanoscale defect-inspection tool. As shown in Figure 2B and C and (Figure S5), the line patterns containing defects exhibited a larger LER and shorter correlation length, which is consistent with the simulated data. In particular, the line patterns containing bridges, foreign nanoparticles, or gaps/breaks showed a larger LER and shorter correlation length, suggesting the existence of defects. Therefore, we could demonstrate the feasibility of STORM-based nanoscale roughness metrology for nanopatterns in semiconductor wafers.

### 2.4 Edge roughness analysis for STORM images of cellular structures

**Figure 3:**
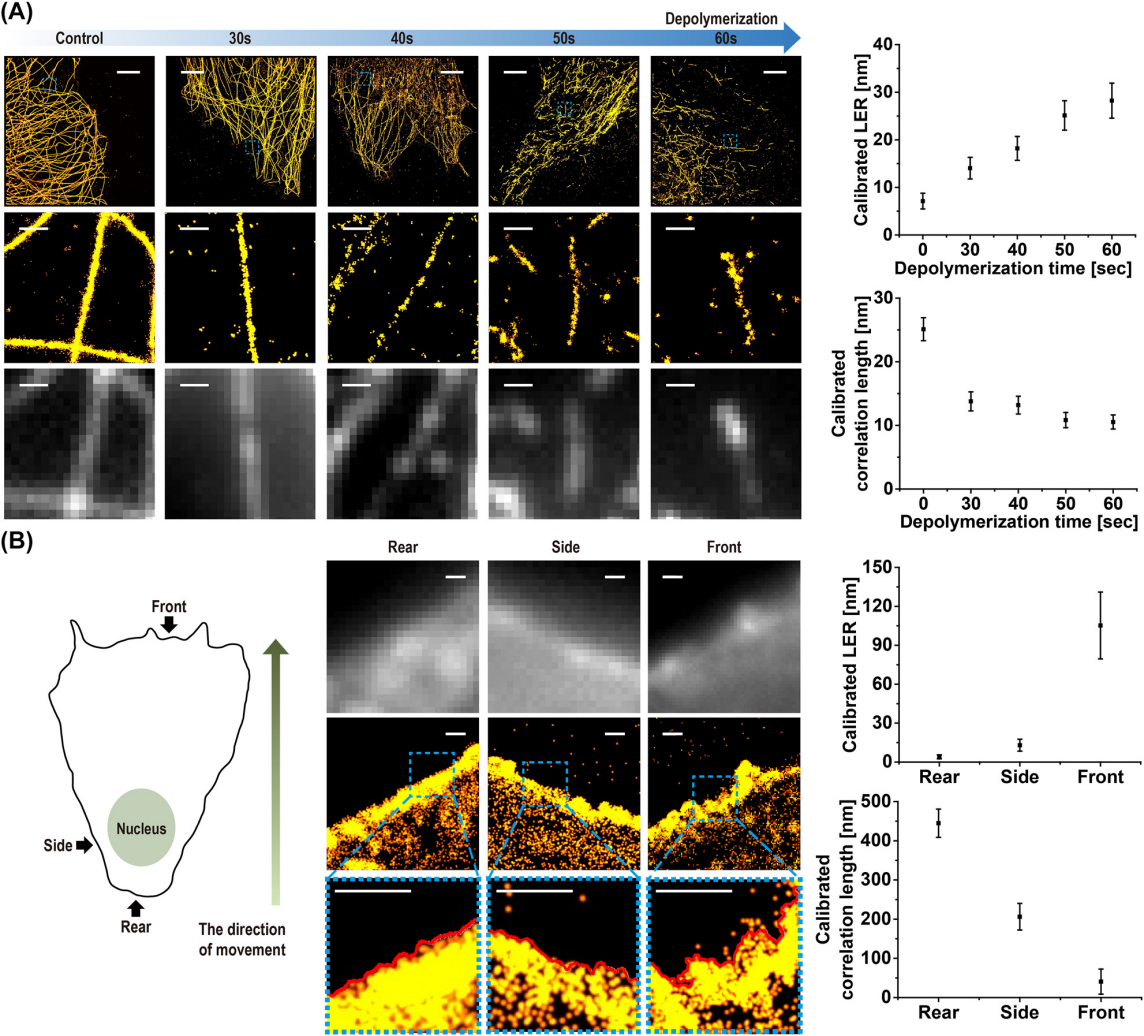
Roughness measurement from STORM images of cellular structures. (A) (Top) Super-resolution STORM images of microtubules during depolymerization process. (Middle) Magnified images of the blue dashed boxes in each top image. (Bottom) Diffraction-limited fluorescence images of the same area (LER and correlation length measured and calibrated from microtubule filaments in STORM images during depolymerization process are shown on the right). (B) Super-resolution STORM images (middle) and their magnified images (bottom) of the blue dashed boxes of cellular membrane stained by Nile red dye. Diffraction-limited fluorescence images of the same area are shown on the top for a comparison. (LER and correlation length measured and calibrated from cell edge boundary for different locations (front, side, and rear) are shown on the right.) Scale bar: 5 μm in top images of (A) and 500 nm in middle and bottom images of (A) and (B) (*n* = 20, mean ± SD).

Next, we applied our roughness analysis to STORM images of various cellular structures, including microtubule filaments and cell membranes. In particular, the measurement of cell membrane edge roughness is considered significant because it can provide insights into the structure and function of cell membranes as well as the status of the cell, such as cancer development or exposure to pollutants [34]. For STORM images of the cell, we used the following rendering parameters, each of which is within its optimal range obtained from the simulation data: localization density = 8 × 10^3^/μm^2^; localization size = 5 nm; background level = 50/μm^2^; localization precision = 8.5 nm. We first tested whether our edge roughness analysis could detect changes in the microtubule filaments during the depolymerization process. To depolymerize the microtubules, we treated the COS-7 cells with nocodazole and performed STORM imaging at different treatment time points. As shown in Figure 3A, our roughness measurements successfully detected nanoscale changes in the microtubule filament edge roughness, which were not clearly noticeable in the diffraction-limited images.

Next, we tested our method for measuring cell-edge roughness to understand its heterogeneity depending on its location, such as the front, side, and rear parts of a moving cell. To visualize nanoscale cell membrane roughness, we stained the COS-7 cells with a membrane dye, Nile red, and performed STORM imaging. As shown in Figure 3B, the cell membrane roughness was clearly observed in the STORM images. Because the overall cell membrane shape is not linear, we fitted the cell membrane edge to an eighth-degree polynomial to remove the general low-frequency cell shape and then calculated the deviation from this fitted polynomial curve, as previously reported [35]. For a better fit to the cell shape, the cell edge with a short distance (∼1–2 μm) was fitted with a polynomial curve, which showed a reasonable cell shape. To investigate the heterogeneity of cell edge roughness in a moving cell, we divided the entire cell into three parts (front, side, and rear) with respect to the moving direction and measured the LER and correlation length. The part in the direction of the cell motion was designated as the front part of the cell, whereas the part opposite the direction of the cell motion was designated as the rear part of the cell. The side part of the cell is the part that is perpendicular to the direction of the cell’s motion. Interestingly, the LER and correlation length (CL) increased from the rear (LER = 12 nm, CL = 409 nm) to the front (LER = 109 nm, CL = 37 nm) of the cell. This can be explained by the differences in the frictional force and interaction with the environment. For example, the front part of the cell experiences the most frictional force and is, therefore, most likely to interact with its environment by forming filopodia at the edge, resulting in a large roughness. In contrast, the rear part of the cell experiences the least frictional force and is most likely to be trailing behind the cell, resulting in a small roughness, whereas the side part of the cell could experience less frictional force than the front part, but still experiences some friction, showing a medium roughness. Such an increase in the front roughness was also observed in a previous study [36]. We also found that the result of our roughness measurement was consistent with the previously reported value [37] as well as with the measurements from SEM and AFM images (Figure S6). Although the AFM was previously used for analyzing the cell roughness, a slow scan rate is required to measure nanoscale roughness in a similar way to the SEM imaging. Also, this often necessitates splitting a single cell into multiple parts for imaging due to the small imaging area for nanoscale imaging, limiting high-throughput analysis. In addition, there is a possibility of dragging the cell membrane during tip scanning, which could damage the cell membrane structure. In contrast, our method can measure highly accurate nanoscale roughness from a one-time STORM imaging of a whole cell if the cell size is within a field of view (33 × 33 μm), allowing high-throughput analysis without damaging the cell structure. Therefore, we demonstrate the feasibility of STORM imaging and roughness analysis as an alternative method for nanoscale cell-edge roughness measurements.

## 3 Conclusions

Here, we report new methods for nanoscale edge structure analysis method for the SMLM images, in terms of the LER and PSD, which have been absent. From the simulated SMLM data, we first investigated the effect of point properties, such as the localization precision, density, size, and background level on the roughness measurement, and then used this effect to estimate the roughness of the real structure. Using these correlations based on simulation data as calibration, we successfully demonstrated roughness measurements from the experimental SMLM images of actual samples, including the semiconductor line patterns, cytoskeletal structures, and cell membrane surfaces. We also demonstrated that our methods can be used for detecting defects on a semiconductor wafer, as well as distinguishing the nanoscale differences in cell membrane roughness.

Although our method demonstrated the imaging and analysis of two-dimensional edge roughness in this study, we expect that it can be extended to images and analyses in three dimensions. While the accuracy of roughness measurements in the axial direction could be lower than that in the lateral direction due to the relatively lower resolution in the axial direction by at least a factor of two, three-dimensional nanoscale roughness analysis would enhance structural understanding.

We note that the method reported here is complementary to, but not a replacement for, previously developed coordinate-based methods. However, our novel approach for nanoscale edge roughness analysis from the SMLM images is anticipated to open the door for further characterization of various edge structures from STORM images, which have not been previously analyzed, and provide new valuable information about a target.

## 4 Methods

### 4.1 Simulated STORM image generation

#### 4.1.1 Semiconductor nanopattern image generation

To simulate the theoretical STORM images with various localization parameters for a given structure, the simulated STORM images were generated using MATLAB (MathWorks, Inc. R2021a). Initially, we defined the key rendering parameters for the simulated STORM images to describe the localization distributions of true line structures. The length and width of the true line structure were set to 1 μm and 450 nm, respectively. When multiple line structures were generated, the pitch between the line patterns was fixed at 900 nm. These values were selected to compare the simulated STORM images with the experimental STORM images of the tested real semiconductor line structures. To investigate the effect of the localization parameters on the roughness measurement from the STORM images, we tested various localization precisions, densities, sizes, and background localization densities. For example, we tested a localization density in the range 0.5 × 10^3^–31.0 × 10^3^/μm^2^ to create theoretically simulated STORM images, which were consistent with the observed localization density from the experimental STORM image data of semiconductor wafers. The localization density can vary depending on the number of photo-switching cycles of the fluorophore, which was set to 16 in this study because the number of switching cycles is known to be ∼16 for a mercaptoethylamine (MEA)-based imaging buffer [10]. When creating the simulated STORM images from the fluorophore distribution, increments in the *x*- and *y*-axes were determined based on the localization density. As a localization precision parameter, we used the full width at half maximum (FWHM) of the distribution of single molecule localization, which reflects the resolution characteristics of the microscope and determines the distribution of single molecule localization [38]. We tested this FWHM in the range 5–50 nm to create theoretically simulated STORM images, which were also consistent with the observed values from the experimental STORM images. For the localization size, we used the same sizes of localizations by applying the uniform Gaussian peaks and the diameter of a localization rendered in the image was based on a size in this study. We tested localization sizes in the range 4–38 nm, which is a reasonable value in the experimental STORM images. To resolve each localization size in this range, we used a pixel size of 3 nm to visualize the nanostructure. We determined that a larger pixel size resulted in significant roughness measurement errors, as expected (Figure S7). As the last localization parameter, we adjusted the background localization density from 0 to 1.0 × 10^3^/μm^2^ as randomly scattered background signal across the entire area. This background localization density range was chosen based on the observations from the experimental STORM images. The resulting simulated STORM image, based on the given localization parameters, was visualized as a scatterplot image with dimensions of 300 × 300 pixels and a scale bar.

#### 4.1.2 Semiconductor nanopattern defect image generation

To generate simulated STORM images for semiconductor line patterns with various defects, we introduced and visualized various types of localized defects, such as nanoparticles, bridges, and breaks on the repetitive line structure in a manner similar to the line pattern generation described earlier. In this test, we fixed the rendering parameter values as the measured value from the experimental STORM images of semiconductor line patterns, which were localization precision of 10.6 nm, localization density of 4.0 × 10^3^/μm^2^, localization size of 5 nm, and background level of 0/μm^2^. The locations of these defects were randomly determined within a repeated line pattern such that they could appear either on the left or right side of the line pattern. The size and morphology of the defects can also be fine-tuned numerically by varying the code parameters. Finally, the resulting wafer image was saved at a resolution of 300 × 300 pixels for an area of 3 μm × 1 μm to preserve the complex details and localization of the defect.

### 4.2 Edge boundary identification from STORM and SEM images

To identify the edge boundaries in the STORM images, we utilized a custom-written MATLAB code (MathWorks, Inc. R2021a). In this code, a selected STORM image was first converted to grayscale using a simple rgb2gray conversion in MATLAB. The transformed grayscale image was then subjected to a series of image preprocessing operations, including erosion and morphological reconstruction, for reasonable edge identification (detailed information can be found in Supplementary Material). These steps ensure the removal of significant empty parts between the localizations in the STORM semiconductor image, thereby allowing for reasonable edge identification. Upon verifying the absence of such empty parts, a well-known method for edge detection, the Canny algorithm, was employed. By adjusting various parameters, such as the threshold and direction, the Canny algorithm could effectively identify the edge boundaries in the STORM semiconductor image. For example, the Canny method employs dual thresholds on the gradient: a high threshold for low edge sensitivity and a low threshold for high edge sensitivity [39]. Pixels with a gradient higher than the high threshold are considered edges, while those with a gradient below the low threshold are rejected. In our analysis, we adopted 0.1 and 0.6 as the low and high thresholds, respectively. For the direction parameter, we used the vertical orientation.

In the edge identification analysis from the AFM and SEM images, the line detection process is more straightforward because the AFM and SEM images are inherently grayscale and do not have gaps between the data points. The Canny algorithm was applied directly to the original AFM and SEM image to facilitate prompt line detection and edge boundary identification.

The resulting edges identified from the STORM and SEM images were superimposed on the original image to check whether our analysis detected the edges reasonably. The resulting high-resolution images were saved for further analysis.

### 4.3 Line-edge roughness (LER) and power spectral density (PSD) analysis

For the LER analysis, high-resolution 2D projections of 3D STORM images of the wafer with 2048 × 2048 pixels and SEM images with 1280 × 960 pixels were analyzed using the custom-written MATLAB codes (MathWorks, Inc.). We first selected the edge boundary of length 1 μm for the semiconductor line pattern and 1 μm for the cell membrane boundary. The line length can be freely adjusted using the drawrectangle function and visualized as a new image. We found that the analyzed length does not affect the roughness measurement as long as the edge has a reasonable line structure (Figure S8). To investigate the dependence of the cell membrane roughness on its location, the cell membrane boundary was divided into three parts with respect to the direction of cell movement: front, side, and rear. The LER is defined as the deviation of the edge from the best-fit central line and can be calculated as three times the standard deviation of the edge. Thus, the edge of the selected line in the array was first detected using the Canny algorithm. Among commonly used edge detection algorithms implemented in MATLAB, such as Canny, Sobel, and Laplacian of Gaussian (LoG), the Canny edge detection algorithm was selected as it enables the most accurate edge detection. Sobel results in disconnected edge detection owing to the absence of a noise reduction step, and LoG demonstrates smoother edge detection with the loss of nanoscale roughness information for our preprocessed STORM images (Figure S9). Next, the central line was then obtained using a linear fit for the detected edge of the semiconductor line pattern or polyfit with an eighth-order function for the detected edge of the cell membrane boundary. The test of the polynomial fitting with various degrees for cell membrane showed that the eighth-degree polynomial fitting exhibits the best fitting compared to the lower-degree polynomial fitting for our STORM images of cell membranes to remove the general low-frequency cell shape (Figure S10). To identify the central line from the detected edge, the detected edge was aligned vertically for the following fitting. Next, to calculate the standard deviation of the detected edge from the fitted central line, the optimal spacing ∆*x* with *N* grid points was selected as a grid size as follows:

(1) L=NΔx

where *L* is the length of the detected edge.

For example, we used the spacing of 3 nm for the 1 μm length analysis. With this optimal spacing value, the edge displacement *d*(*x*) was calculated at position *x*. Then, the standard deviation (*σ*) of the detected edge from the fitted central line was calculated for LER analysis. As reported previously [25], the LER was calculated to be thrice the standard deviation (*σ*) of the edge.

(2) LER=3σ

This calculation was also used for the PSD analysis. The edge displacement *d*(*x*) was calculated at position *x*, which was used to obtain the PSD curve after the Fourier transformation as follows [24]:

(3) $$\text{PSD}\left(f\right)=L\times \left|\text{FFT}\right.{\left.\;\;\left(d\left(x\right)\right)\right|}^{2}$$

where *L* is the length of the detected edge and *f* represents the spatial frequency. To generate the PSD curve, the optimal spacing ∆*x* with *N* grid points was chosen as a grid size.

The PSD curve for each selected edge as a function of frequency was obtained using Fourier transform and averaged to minimize the statistical noise. Next, we attempted to determine the correlation length from the experimentally obtained PSD curves by fitting them with the theoretical PSD curve. The theoretical PSD curve was obtained using the following equation, as previously reported [24]:

(4) $${\text{PSD}}_{\text{biased}}\left(f\right)={\text{PSD}}_{\text{unbiased}}\left(f\right)+{\sigma^{2}}_{\text{noise}}\times L$$

where PSD_biased_ is the PSD measured directly from the STORM image, PSD_unbiased_ is the unbiased PSD measured from the true roughness of the line, *σ*_noise_ is the random error in the detected edge position due to noise in the STORM image, and ∆*L* is the grid size along the length of the line. Briefly, the noise from the edge detection was combined with the inherent roughness of the patterns on the wafer, resulting in a positive bias in the measured roughness. The theoretical unbiased PSD (PSD_unbiased_) generated from the true roughness in one dimension was proposed by Palasantzas (1993) as follows [40]:

(5) $${\text{PSD}}_{\text{unbiased}}\left(f\right)=2\sigma^{2}\xi \frac{\sqrt{\pi }\Gamma \left(H+\frac{1}{2}\right)}{\Gamma \left(H\right)}/\;{\left[1+{\left(2\pi f\xi \right)}^{2}\;\right]}^{\;H+\frac{1}{2}}$$

where, ${\sigma^{2}}_{\text{noise}}$ is a random error detected in the linewidth owing to the noise in the images and edge detection, *σ* is the standard deviation of line-edge, *ξ* is the correlation length, Γ is the gamma function, and *H* is the Hurst exponent. The typical Hurst exponent is 0.5, corresponding to an exponential autocorrelation function; thus, we fixed the Hurst exponent at 0.5, which also showed the best fit with our data [23]. Briefly, Parseval’s theorem states that the integral across the PSD equals the variance *σ* ^2^ [24]. An increase in *σ* increases the offset of the PSD. The correlation length dictates the shift from frequency-independent white noise at low spatial frequencies to decay with 1/*f* characteristics at high frequencies. A greater correlation length results in a less rough edge appearance, even with a constant *σ*. The Hurst exponent influences the slope of decay at higher spatial frequencies. Once the PSD curve was obtained from the selected line in the experimental data, the PSD data were averaged and fitted to the theoretical PSD equations ([4] and [5]) using the lsqnonlin function in MATLAB. Because all the parameters except the correlation length are known, the optimized correlation length can be determined from the fitting.

## Supplementary Material

Supplementary Material Details

## References

[1] Hell S. W., Wichmann J. (1994). Breaking the diffraction resolution limit by stimulated emission: stimulated-emission-depletion fluorescence microscopy. *Opt. Lett.*.

[2] Gustafsson M. G., Agard D. A., Sedat J. W. (1995). *Three-dimensional microscopy: image acquisition and processing II*.

[3] Rust M. J., Bates M., Zhuang X. (2006). Sub-diffraction-limit imaging by stochastic optical reconstruction microscopy (STORM). *Nat. Methods*.

[4] Betzig E. (2006). Imaging intracellular fluorescent proteins at nanometer resolution. *Science*.

[5] Lippincott-Schwartz J., Manley S. (2009). Putting super-resolution fluorescence microscopy to work. *Nat. Methods*.

[6] Park Y. (2022). Polarity nano-mapping of polymer film using spectrally resolved super-resolution imaging. *ACS Appl. Mater. Interfaces*.

[7] Nguyen D. T. (2022). Super-resolution fluorescence imaging for semiconductor nanoscale metrology and inspection. *Nano Lett*..

[8] Jeong U. (2023). Development of highly dense material-specific fluorophore labeling method on silicon-based semiconductor materials for three-dimensional multicolor super-resolution fluorescence imaging. *Chem. Mater.*.

[9] Wöll D., Flors C. (2017). Super‐resolution fluorescence imaging for materials science. *Small Methods*.

[10] Chung J., Jeong U., Jeong D., Go S., Kim D. (2022). Development of a new approach for low-laser-power super-resolution fluorescence imaging. *Anal. Chem.*.

[11] Kim D., Deerinck T. J., Sigal Y. M., Babcock H. P., Ellisman M. H., Zhuang X. (2015). Correlative stochastic optical reconstruction microscopy and electron microscopy. *PloS One*.

[12] Hyun Y., Kim D. (2023). Recent development of computational cluster analysis methods for single-molecule localization microscopy images. *Comput. Struct. Biotechnol. J.*.

[13] Sengupta P., Jovanovic-Talisman T., Skoko D., Renz M., Veatch S. L., Lippincott-Schwartz J. (2011). Probing protein heterogeneity in the plasma membrane using PALM and pair correlation analysis. *Nat. Methods*.

[14] Levet F. (2015). SR-Tesseler: a method to segment and quantify localization-based super-resolution microscopy data. *Nat. Methods*.

[15] Hartley J. M. (2015). Super‐resolution imaging and quantitative analysis of membrane protein/lipid raft clustering mediated by cell‐surface self‐assembly of hybrid nanoconjugates. *ChemBioChem*.

[16] Andronov L., Lutz Y., Vonesch J.-L., Klaholz B. P. (2016). SharpViSu: integrated analysis and segmentation of super-resolution microscopy data. *Bioinformatics*.

[17] Chan C. Y., Pedley A. M., Kim D., Xia C., Zhuang X., Benkovic S. J. (2018). Microtubule-directed transport of purine metabolons drives their cytosolic transit to mitochondria. *Proc. Natl. Acad. Sci. U.S.A.*.

[18] Williamson D. J. (2020). Machine learning for cluster analysis of localization microscopy data. *Nat. Commun.*.

[19] Marenda M., Lazarova E., van de Linde S., Gilbert N., Michieletto D. (2021). Parameter-free molecular super-structures quantification in single-molecule localization microscopy. *J. Cell Biol.*.

[20] Bunday B. D., Bishop M., Villarrubia J. S., Vladar A. E. (2003). CD-SEM measurement line-edge roughness test patterns for 193-nm lithography. *Metrology, inspection, and process control for microlithography XVII*.

[21] Antonio P. D., Lasalvia M., Perna G., Capozzi V. (2012). Scale-independent roughness value of cell membranes studied by means of AFM technique. *Biochim. Biophys. Acta – Biomembr.*.

[22] Orji N. G., Vorburger T. V., Fu J., Dixson R. G., Nguyen C. V., Raja J. (2005). Line edge roughness metrology using atomic force microscopes. *Meas. Sci. Technol.*.

[23] Mack C. A. (2013). Generating random rough edges, surfaces, and volumes. *Appl. Opt.*.

[24] Siefke T. (2018). Line-edge roughness as a challenge for high-performance wire grid polarizers in the far ultraviolet and beyond. *Opt. Express*.

[25] Mack C. A., Lorusso G. F. (2019). Determining the ultimate resolution of scanning electron microscope-based unbiased roughness measurements. I. Simulating noise. *J. Vac. Sci. Technol. B: Nanotechnol. Microelectron.*.

[26] Shroff H., Galbraith C. G., Galbraith J. A., Betzig E. (2008). Live-cell photoactivated localization microscopy of nanoscale adhesion dynamics. *Nat. Methods*.

[27] Nyquist H. (1928). Certain topics in telegraph transmission theory. *Trans. Am. Inst. Electr. Eng*.

[28] Shannon C. E. (1949). Communication in the presence of noise. *Proc. IRE*.

[29] Zhuang X. (2009). Nano-imaging with STORM. *Nat. Photonics*.

[30] Cutler C. (2018). Utilizing roughness power spectral density variables to guide resist formulation and understand impact of frequency analysis through process. *J. Photopolym. Sci. Technol.*.

[31] Diaz C. H., Tao H.-J., Ku Y.-C., Yen A., Young K. (2001). An experimentally validated analytical model for gate line-edge roughness (LER) effects on technology scaling. *IEEE Electron Device Lett*..

[32] Patterson K. (2001). Experimental determination of the impact of polysilicon LER on sub-100-nm transistor performance. *Metrology, inspection, and process control for microlithography XV*.

[33] Asenov A., Kaya S., Brown A. R. (2003). Intrinsic parameter fluctuations in decananometer MOSFETs introduced by gate line edge roughness. *IEEE Trans. Electron Devices*.

[34] Klähn M., Zacharias M. (2013). Transformations in plasma membranes of cancerous cells and resulting consequences for cation insertion studied with molecular dynamics. *Phys. Chem. Chem. Phys.*.

[35] Gesper A., Wennmalm S., Hagemann P., Eriksson S.-G., Happel P., Parmryd I. (2020). Variations in plasma membrane topography can explain heterogenous diffusion coefficients obtained by fluorescence correlation spectroscopy. *Front. Cell Dev. Biol.*.

[36] Rapin G. (2021). Roughness and dynamics of proliferating cell fronts as a probe of cell–cell interactions. *Sci. Rep.*.

[37] Chang C.-H., Lee H.-H., Lee C.-H. (2017). Substrate properties modulate cell membrane roughness by way of actin filaments. *Sci. Rep.*.

[38] Dempsey G. T., Vaughan J. C., Chen K. H., Bates M., Zhuang X. (2011). Evaluation of fluorophores for optimal performance in localization-based super-resolution imaging. *Nat. Methods*.

[39] Canny J. (1986). A computational approach to edge detection. *IEEE Trans. Pattern Anal. Mach. Intell.*.

[40] Palasantzas G. (1993). Roughness spectrum and surface width of self-affine fractal surfaces via the K-correlation model. *Phys. Rev. B*.

