# Supplementary material for: Edge roughness analysis in nanoscale for single-molecule localization microscopy images
Source: Nanophotonics. 2024 Jan 4;13(2):195–207. doi: 10.1515/nanoph-2023-0709 (PMC11501732; doi:10.1515/nanoph-2023-0709)
Supplement: Supplementary file 1 — Supplementary Material Details [file j_nanoph-2023-0709_suppl_001.pdf]

Supplementary material

Edge roughness analysis in nanoscale for single-molecule localization microscopy images

Uidon Jeong, Ga-eun Go, Dokyung Jeong, Dongmin Lee, Min Jeong Kim, Minjae Kang, Namyoon Kim, Jaehwang Jung, Wookrae Kim, Myungjun Lee, Doory Kim*

1. Experimental Section

1.1 Sample preparation for semiconductor imaging

For semiconductor imaging, silica nanopatterned arrays fabricated on a silicon wafer were labeled with fluorophores in the same manner as previously reported.[1] Briefly, the wafers were first sonicated in acetone for 15 min at room temperature (RT) as a washing step and then dried. For silica-specific labeling, wafers were incubated for 15 min in 0.01% poly-L-lysine (PLL) (P4707; Sigma-Aldrich) solution and rinsed twice with Dulbecco's phosphate-buffered saline (DPBS). To label the fluorophores on the PLL-coated wafer, the PLL-labeled wafer was incubated in Alexa Fluor 647-conjugated bovine serum albumin (BSA) (A34785; Sigma-Aldrich) solution with a concentration of 5 μg mL^{-1} in 0.1 M glycine (077-00735; Wako Pure Chemical Industries, Ltd) buffer at pH 11 for 60 min at RT. For Si-specific labeling, the wafers were immersed in a 5% Nafion solution (510211; Sigma-Aldrich) for 15 min at RT and then washed twice with DPBS. To achieve a homogeneous Nafion coating, the wafers were spin-coated with a 5% Nafion solution at 4,500 rpm for 70 s. After two washes with DPBS, the

Nafion-coated wafer was labeled with fluorophores using the Alexa Fluor 647-conjugated BSA solution of concentration 5 μg mL^{-1} in a 0.1 M glycine buffer at pH 3. Upon completion of either silica- or silicon-specific labeling, immediate imaging was performed using an imaging buffer. This buffer was composed of 100 mM mercaptoethylamine (MEA) (30070; Sigma-Aldrich), 5% (w/v) glucose (G8270; Sigma-Aldrich), and oxygen-scavenging enzymes [0.5 mg mL^{-1} glucose oxidase (G2133; Sigma-Aldrich) and 38 μg mL^{-1} catalase (C3515; Sigma-Aldrich)] in DPBS at pH 8.5, and is known to generate the best photoswitching properties of dyes at high laser power conditions.[2] To minimize the spherical aberration arising from the inverted imaging geometry, 60% (w/v) sucrose was added to adjust the refractive index to 1.45.

1.2 Sample preparation for cell imaging

For cell imaging, COS-7 cells were cultured on glass-bottom confocal dish and grown at 37 ℃ and 5% CO_2 for 1–2 d in culture media. The cells were immunolabeled in the same manner as previously reported.[3] Briefly, seeded cells were washed using DPBS and fixed with 3% (v/v) paraformaldehyde (PFA) (15714; Electron Microscopy Sciences) and 0.1% (v/v) glutaraldehyde (GA) (16020; Electron Microscopy Sciences) in DPBS for 10 min at RT. The fixed cells were rinsed with DPBS and treated with freshly prepared 0.1% (w/v) $NaBH_4$ (71320; Sigma-Aldrich) in DPBS for 7 min at RT to remove the unreacted aldehyde groups. The reduced cells were permeabilized with 0.25% Triton X-100 for 10 min at RT. After permeabilization, the cells were incubated in blocking buffer 3% (w/v) BSA for 30 min at RT. The cells were stained with a Anti-tubulin primary antibody (ab6160; Abcam) in blocking buffer for 30 min at RT. After washing once with DPBS, the cells were labeled with Alexa Fluor 647-conjugated goat anti-rat secondary antibody (A21247; Invitrogen) in blocking buffer for 60 min at RT. The cells were post-fixed with 2% (v/v) PFA and 0.05% (v/v) GA in DPBS for 10 min at RT. Finally, the samples were either stored in DPBS at 4 ℃ or immersed in a STORM imaging buffer if

they need to be imaged immediately. The STORM imaging buffer was prepared using 100 mM MEA, 5% (w/v) glucose and oxygen-scavenging enzymes (0.5 mg mL^{-1} glucose oxidase and 38 μg mL^{-1} catalase) in DPBS at pH 8.5).

For the microtubule depolymerization experiment, 1 μM nocodazole (M1404; Sigma-Aldrich) solution in Dimethyl sulfoxide (DMSO) was added for 30, 40, 50, and 60 s before the fixation at RT. After treatment with nocodazole, the cells were rinsed with DPBS for 5 min and fixed with 3% (v/v) PFA and 0.1% (v/v) GA in DPBS for 10 min at RT. The fixed cells were reduced with 0.1% (w/v) $NaBH_4$ in DPBS, labeled with a dye-conjugated antibody, and post-fixed as described earlier.[3]

For the cell membrane staining, the fixed cells were washed with DPBS and reduced with 0.2% (w/v) $NaBH_4$ in DPBS for 6 min at RT. After washing once briefly with DPBS, the samples were stained with 10 nM Nile Red (415711000; Acros Organics) solution in DPBS and imaged using a STORM, in the same manner as previously reported.[4]

1.3 STORM imaging

All the STORM images were acquired using a custom-built STORM setup that was specially designed with an inverted microscope and 1.49 NA 100x oil immersion objective lens (CFI SR HP Apo TIRF; Nikon), as reported.[1] Lasers with wavelengths of 561 nm (OBIS; Coherent) and 647 nm (OBIS; Coherent) were employed for fluorophore excitation, whereas a 405 nm (OBIS; Coherent) laser was used for fluorophore reactivation. The laser illuminated the sample by passing through the back port of the microscope body, and the angle of incidence was adjusted for total internal reflection fluorescence (TIRF) illumination. During imaging, illumination intensity was consistent as 100 mW at 561 nm, 120 mW at 647 nm, and 0.5–1 mW at 405 nm. The CRISP Autofocus system (ASI) was used to control the focus by detecting the

IR beam reflected at the sample-liquid interface. The emitted fluorescence was filtered using bandpass emission filters (LF408/488/561/635-B; Semrock) and images were acquired using an electron-multiplying charge-coupled device (EMCCD) camera (iXon Ultra 888; Andor). Images were recorded at a frame rate of 60–100 Hz over an area of 33 μm x 33 μm and 40,000–70,000 frame images were collected. To determine the lateral position of each molecular centroid, the point spread function (PSF) was fitted with a Gaussian function as previously described.[5] For the axial position, a cylindrical lens (LJ1144RM-A; Thorlabs) was placed in the fluorescence detection path to obtain the z-position information based on the ellipticity of the PSF. The centroids were then collected as localizations and drift-corrected for a final STORM image in both the lateral and axial dimensions.

1.4 Image reconstruction and preprocessing operations

Following the recording of the STORM movie, each point spread function generated from a single fluorophore emission was fitted with a two-dimensional Gaussian peak. The centroid of each fitted Gaussian peak was subsequently determined to represent the localization position, as outlined in previous reports.[6] Briefly, the fluorescence images of a raw STORM movie within regions where a single switch was active were subject to analysis for localization. The fluorescence intensities of the single-molecule point spread function (PSF) above the background height were then fitted to a continuous ellipsoidal Gaussian function using nonlinear least-squares regression, as previously detailed.[6] To ensure accurate identification of single molecules, it was crucial to use a high threshold for the single-molecule PSF for mitigating false identifications arising from background noise. Optimal threshold determination was recognized as dependent on varying background levels across setups and settings. Following the identification of localizations in each frame, a collection process ensued, followed by drift correction to reconstruct the final STORM images. For each localization,

single-molecule information, such as centroid positions for x and y, fluorescence intensity, the number of photons per switching cycle, on-time, and background intensity, was obtained. For rendering each localization, uniform Gaussian peaks were applied to use the same sizes of localizations, where the diameter of the localization rendered in the image was used as the localization size.

In the context of localization precision measurements, approximately 20 clusters were initially aligned based on their center of mass. This alignment facilitated the determination of the distribution of deviations from the centroid positions, with the resulting histogram subsequently fitted to a two-dimensional Gaussian function. The full width at half maximum (FWHM) and the standard deviation extracted from the Gaussian function served as the spatial resolution and localization precision, respectively, for the STORM image under each condition.

For effective edge detection, erosion and dilation operations were performed during preprocessing. First, erosion was performed to remove noise signals using the imerode function in MATLAB. The erosion operation uses a structured element kernel composed of 0 and 1. This kernel moves over each pixel of the STORM image by selecting areas to be eroded. We used a disk-shaped kernel that marks a pixel as 1 only when it exactly matches the entire pixel. Unmatched areas are marked as 0, thus eroding specific pixels. We determined that erosion was effective in removing noise arising from background localization in STORM images, as it eliminated small shapes. Next, we employed a dilation operation as a morphological reconstruction, which is the opposite of the erosion process, to fill the empty areas in the STORM images. The imdilate function in MATLAB was used for dilation. It uses a structured element kernel composed of 0 and 1, but marks a pixel as 0 when the kernel exactly matches the entire pixel. When it did not exactly match, it was marked as one. We determined that the dilation effectively filled the empty spaces in the detected lines. In both erosion and dilation operations, the size parameter of the kernel can be adjusted to vary between 1 and 4.

After image preprocessing step, the edge was detected by the Canny algorithm. The Canny algorithm for edge detection involves a five-step process.[7] First, Gaussian blurring is applied to reduce the noise in the image. The Gaussian kernel used during this process minimizes the noise without damaging the areas of interest in the image. Second, the magnitude and direction of the gradient are calculated for each pixel. The gradient calculated in this step is crucial for the accurate identification of the edges. Third, nonmaximum suppression refines the edges identified in the previous step. This involves inspecting each pixel in the gradient magnitude image and excluding values that are not maxima in their gradient direction, thereby enhancing the detection clarity. Fourth, dual thresholding is performed to distinguish between strong and weak edges. Strong edges have higher thresholds, allowing for the exclusion of edges with lower thresholds. This ensures a more accurate continuity of the edges. Finally, an edge map is created that provides sharp contrast against non-edge areas and visualizes the identified edges.

1.5 SEM imaging

We performed SEM imaging of a semiconductor wafer for comparison with the STORM images. Before the SEM imaging, all the wafers were sonicated in acetone for 15 min and then dried. After drying, the wafers were imaged using an S-4800 FE-SEM (Hitachi) at 5 keV. We tested the scanning rate of 0.03–3 Hz and magnification of 3.0 k–12.0 k to investigate the effect of image noise on the roughness measurement. To compare the STORM images, the same type of wafer was imaged by SEM and SEM images with 1,280 × 960 pixels for an area of 10.2 μm x 7.7 μm (pixel size = 8 nm) were obtained. For SEM imaging of the cell membrane, the sample was prepared in a manner similar to the previous report.[8] Firstly, 12-mm diameter cover glasses were sterilized with 70% (v/v) ethanol (4023-4100; DAEJUNG) and exposed to UV light for 15 minutes. Subsequently, COS-7 cells were cultured on them at 37°C and 5% CO_2

for 1-2 days in DMEM. To stabilize the ultrastructure of cells before further processing, the seeded cells were fixed by adding a solution of 2.5% (v/v) GA in DPBS for 1 day at 4°C. The fixed cells were then washed twice with DPBS to remove excess fixative. The washing process was followed by post-fixation using a 1% (v/v) osmium tetroxide (19152; Electron Microscopy Sciences) aqueous solution for 10 minutes at room temperature. After post-fixation, the sample was briefly rinsed twice with distilled water and dehydrated using a series of diluted ethanol (30%, 50%, 70%, 80%, 90%, and 100% [v/v]) in distilled water. Each step involved immersing the sample in the ethanol solutions for 10 minutes to replace water with ethanol. Following the dehydration process, the sample was treated with hexamethyldisilazane (4079-4105; DAEJUNG) for 15 minutes at room temperature and dried in the air for 3-8 hours. The completely dried sample was mounted on a sample holder using a conductive adhesive, such as carbon tape. To enhance conductivity and reduce charging effects during SEM imaging, the sample was sputter-coated with platinum before imaging. The Pt-coated cells were imaged using an S-4800 FE-SEM (Hitachi) at 10 keV. For nanoscale roughness measurement, we used a scanning rate of 0.1 Hz and magnification of 20 k. SEM images with 1,280 × 960 pixels for an area of 7.0 μm x 5.3 μm (pixel size = 5.5 nm) were obtained.

1.6 AFM imaging

For AFM imaging of the cell membrane, 18 x 18 mm cover glasses were incubated in a 0.01% poly-L-lysine (PLL) solution for 15 minutes to enhance cell adhesion to the substrates. Subsequently, the cover glasses were briefly rinsed with DPBS twice and dried in the air. COS-7 cells were cultured on the PLL-coated substrates and grown at 37°C and 5% CO_2 for 1-2 days in DMEM. The samples were then rinsed with DPBS for 5 minutes three times and fixed with 4% (v/v) PFA for 30 minutes at room temperature. Following fixation, the samples were rinsed with DPBS for 5 minutes. Subsequently, imaging was performed in non-contact mode using an

XE-100 (Park Systems). For nanoscale roughness measurement, we used a scanning rate of 0.5 Hz and magnification of 20 k. AFM images with 256 × 256 pixels for an area of 5 μm x 5 μm (pixel size = 19.5 nm) were obtained.

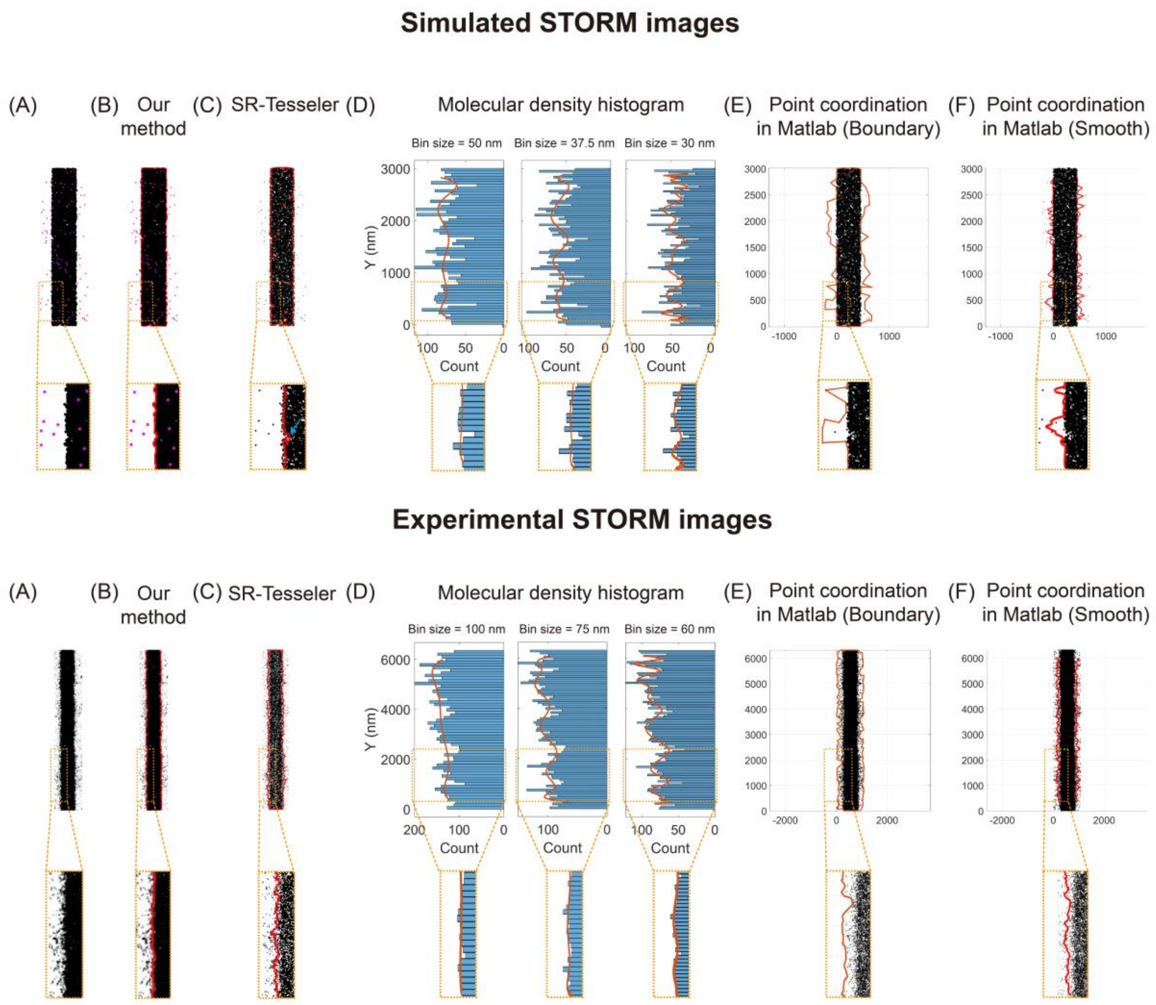

Figure S1: Comparison of the edge detection analysis methods for simulated (top) and experimental (bottom) STORM images. (A) STORM images of a line pattern. Violet: background localization. (B) Localization-rendered image-based edge detection using the Canny algorithm (our method). (C–E) Localization coordinate-based method. (C) SR-Tesseler (D) Molecular density distribution with polynomial fitting (bin size effects are shown together.) (E) Edge trace using boundary function in MATLAB. (F) Edge trace using smooth function in MATLAB.

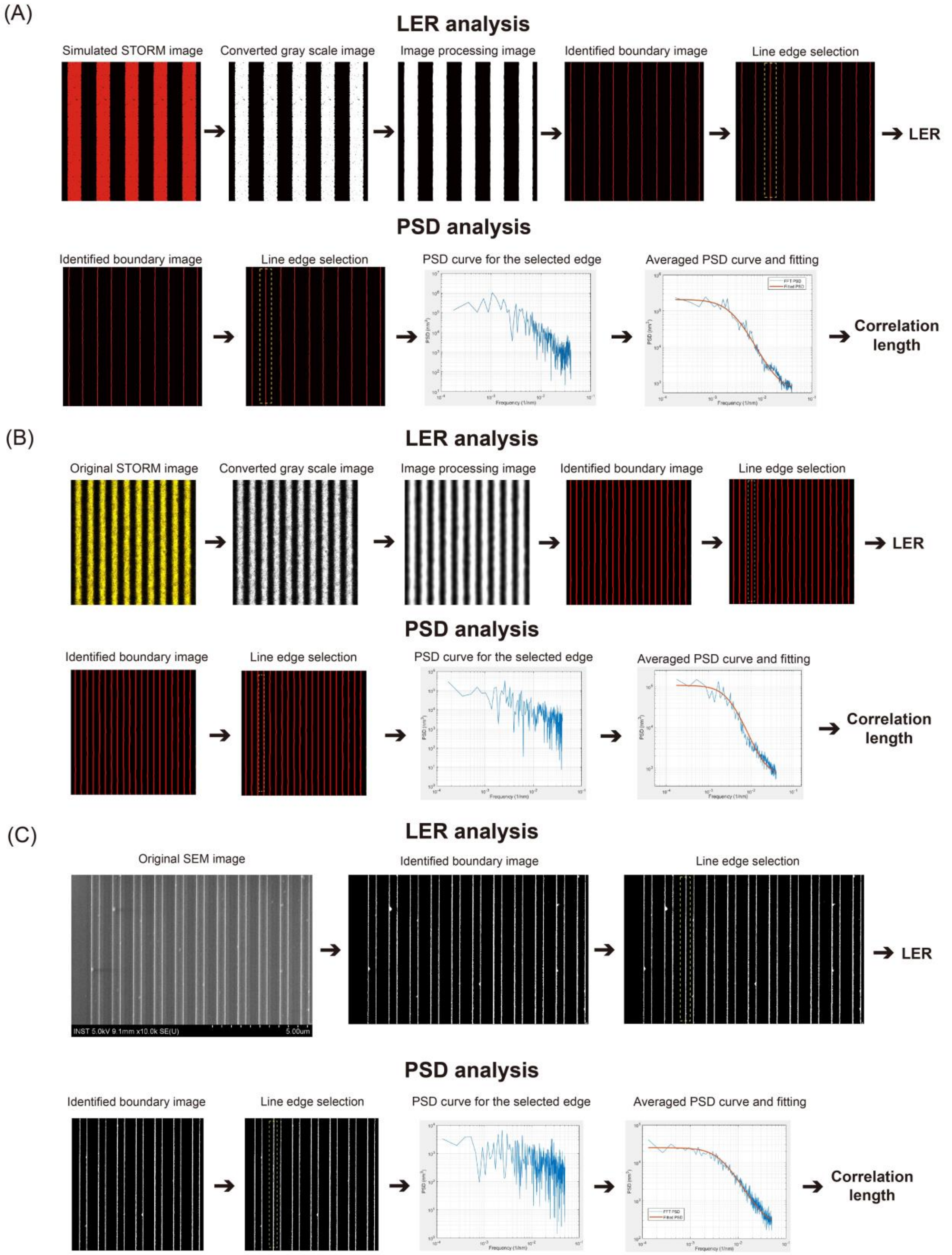

Figure S2: LER and PSD analysis procedure for simulated STORM images (A), experimental STORM images (B), and SEM images (C) of nanopattern arrays.

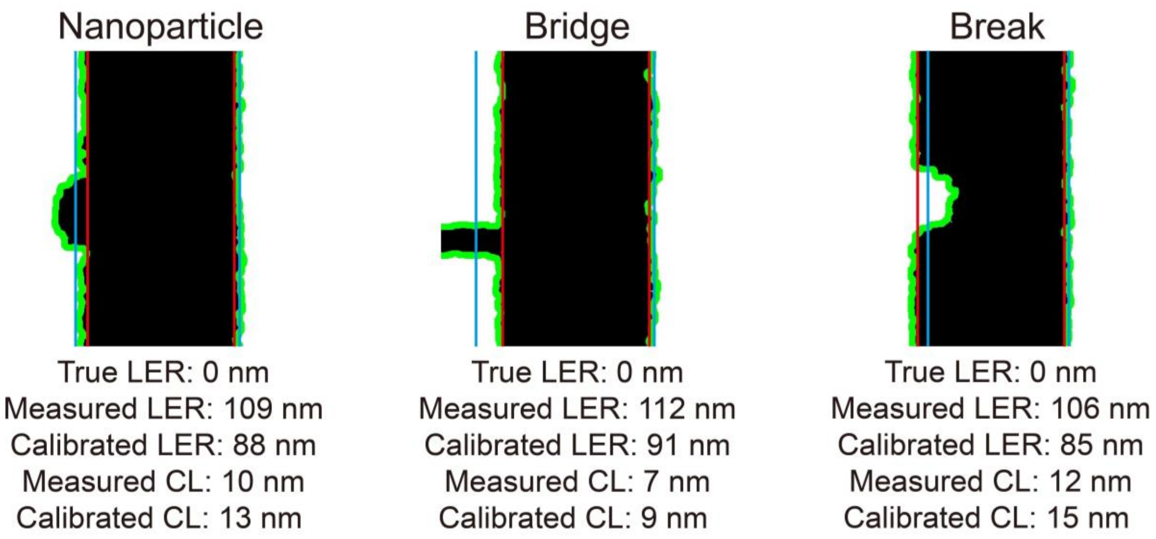

Figure S3: Simulated STORM images of a line pattern (true LER = 0) with various defects, including nanoparticle (top), bridge (middle), and break (bottom), using experimental image parameters (localization precision = 10.6 nm, localization density = $4.0\times10^3/\mu m^2$, localization size = 5 nm in diameter, background localization level = $0/\mu m^2$) (true edge, identified edge and fitted central line for each edge are shown in red, green, and blue, respectively; their measured and calibrated LER value and correlation length are shown on the bottom of each image). (n = 15, mean ± SD)

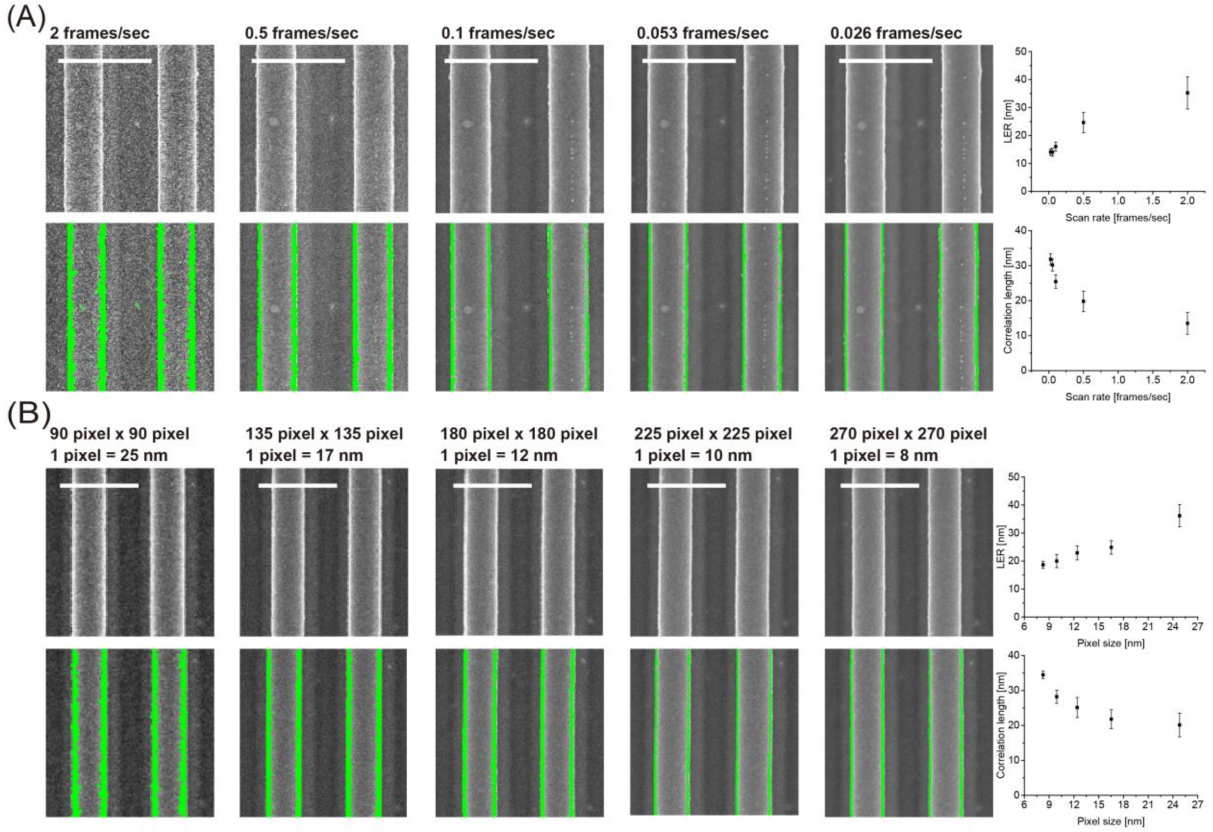

Figure S4: SEM images of semiconductor nanopattern arrays (pitch: 1,000 nm; width: 450 nm) obtained at various scan rates or pixel sizes (magnification) (identified edge is shown in green, and their LER and correlation length measured from PSD curve analysis result are shown on the right). Scale bar: 1 μm. (n = 20, mean ± SD)

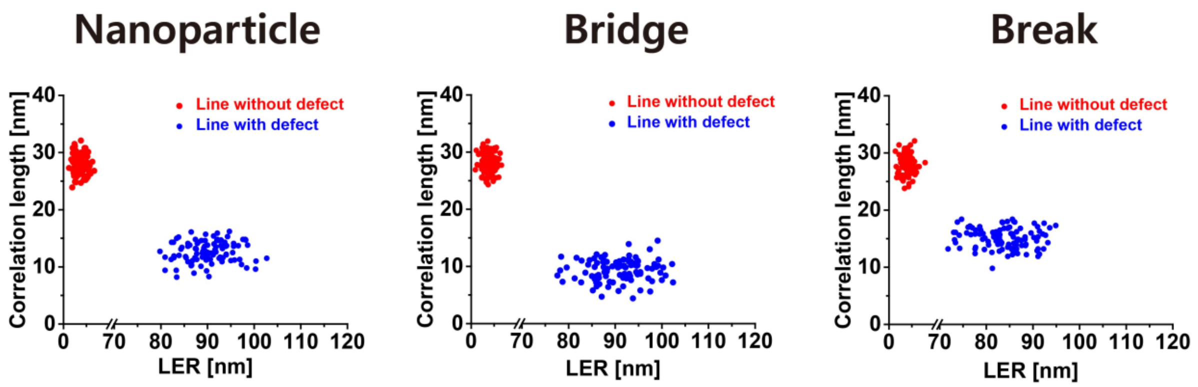

Figure S5: Comparison of the distribution of the LER and correlation length obtained from experimental STORM images of line structures with or without various defects, including nanoparticles, bridges, and breaks. The measured LER and correlation lengths for the line patterns with defects were observed in the distinct range from those of the line patterns without defects, irrespective of the defect type.

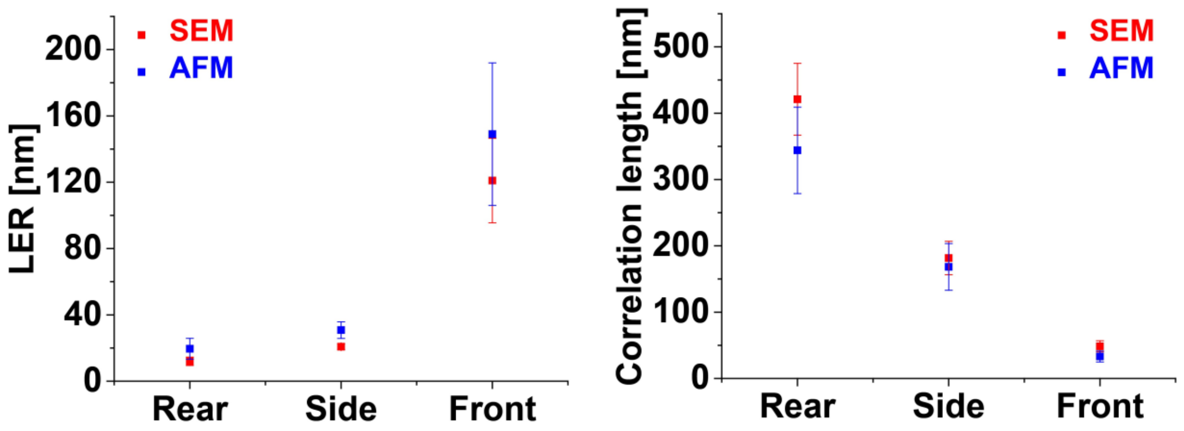

Figure S6: LER and correlation length measured from SEM and AFM images of the cell edge boundaries for various locations (rear, side, and front).

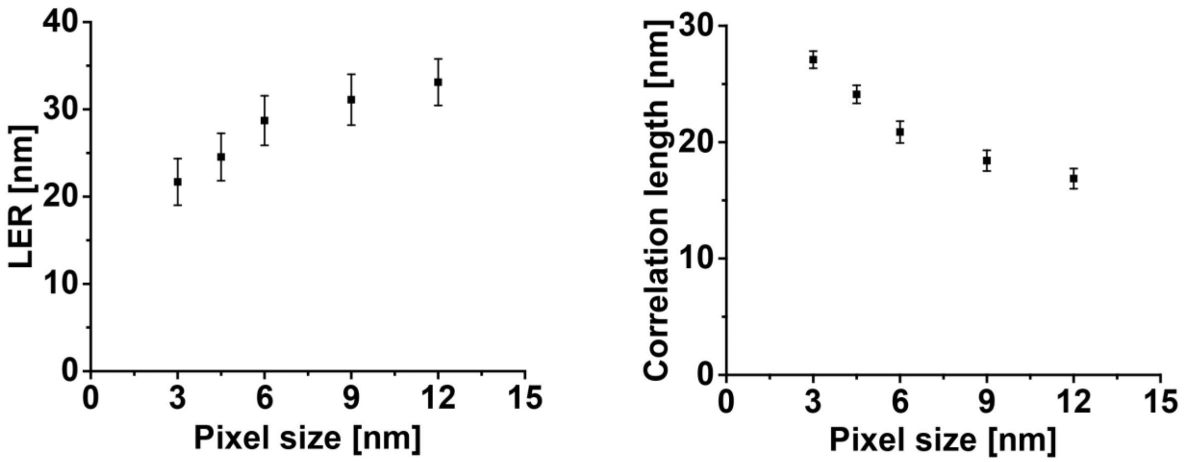

Figure S7: Effect of pixel size in SMLM images on roughness measurements.

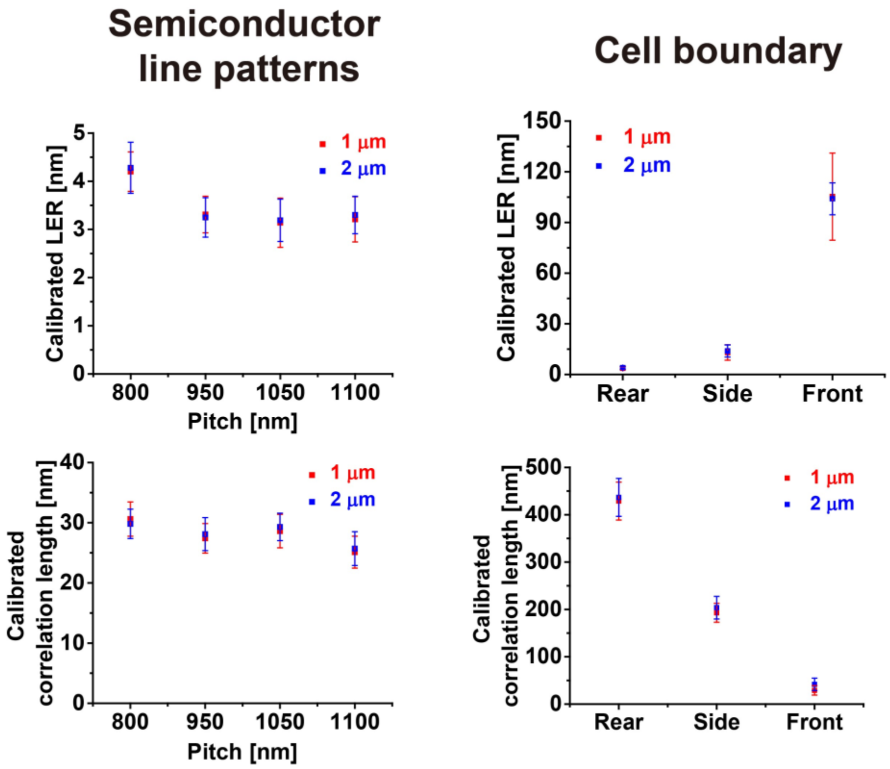

Figure S8: Comparison of the roughness measurements obtained using various analyzed lengths (1 and 2 μm) in STORM images of semiconductors and cell membranes, which does not affect the roughness measurement as long as the edge has a reasonable line structure.

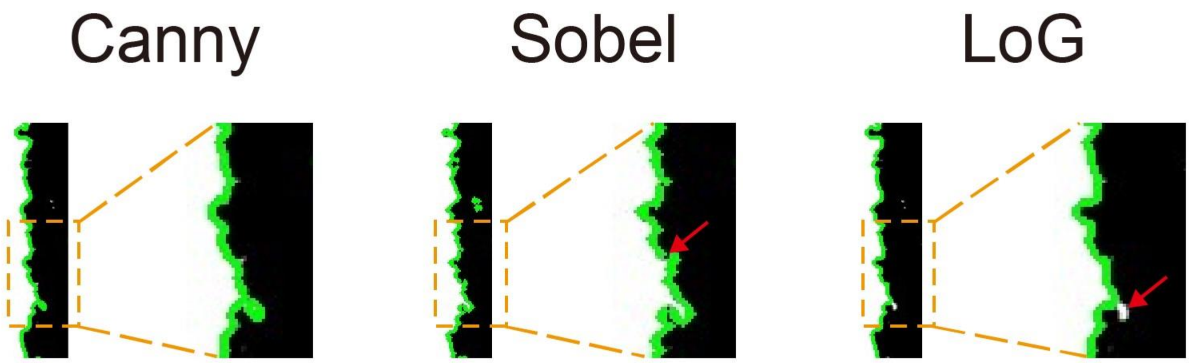

Figure S9: Comparison between different edge detection algorithm. The Canny algorithm is effective in detecting true edges by minimizing the detection of non-edges caused by noise, whereas Sobel exhibits disconnected edge detection and LoG demonstrates smoother edge detection with the loss of nanoscale roughness information.

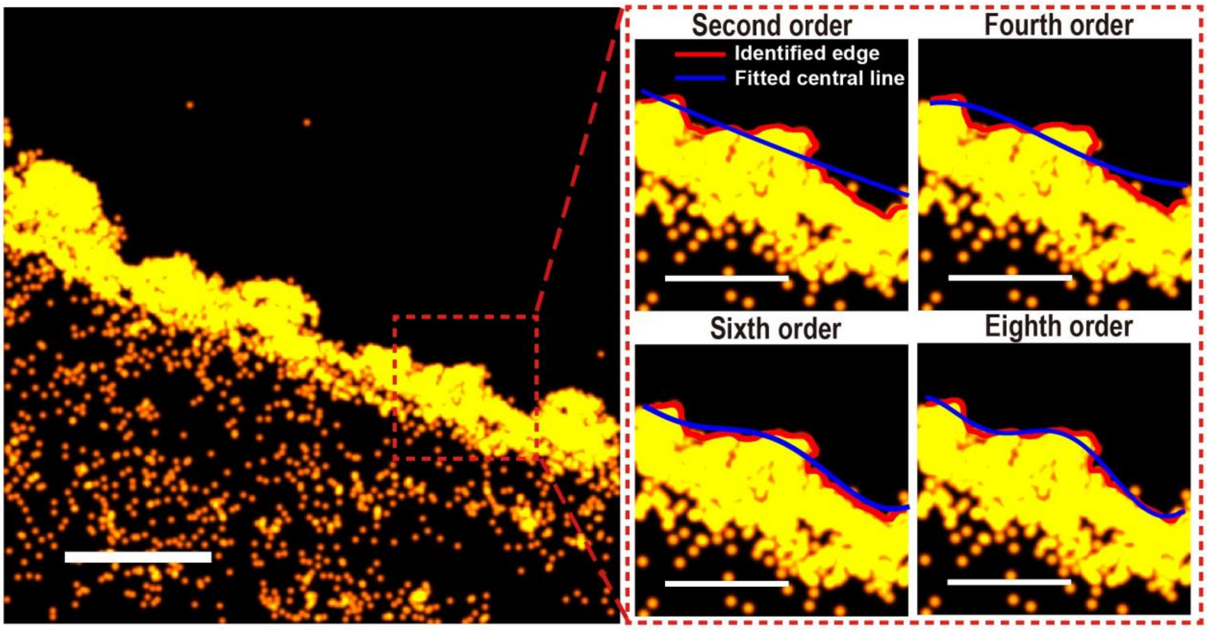

Figure S10: Comparison of fitting the cell membrane edge with different polynomial orders (n=2,4,6,8). The eighth-degree polynomial fitting exhibits the best fitting compared to the lower-degree polynomial fitting. The identified edge and fitted central line for each edge are shown in red and green, respectively. Scale bar: 1 μm in left and 500 nm in right.

References

[1] U. Jeong *et al.*, "Development of Highly Dense Material-Specific Fluorophore Labeling Method on Silicon-Based Semiconductor Materials for Three-Dimensional Multicolor Super-Resolution Fluorescence Imaging," *Chem. Mater.,* vol. 35, no. 14, pp. 5572-5581, 2023

[2] J. Chung, U. Jeong, D. Jeong, S. Go, and D. Kim, "Development of a New Approach for Low-Laser-Power Super-Resolution Fluorescence Imaging," *Anal. Chem.,* vol. 94, no. 2, pp. 618-627, 2022

[3] S. Go *et al.*, "Super-resolution imaging reveals cytoskeleton-dependent organelle rearrangement within platelets at intermediate stages of maturation," *Structure,* vol. 29, no. 8, pp. 810-822. e3, 2021

[4] M. J. Kim *et al.*, "Bacteria detection and species identification at the single-cell level using super-resolution fluorescence imaging and AI analysis," *Biosens. Bioelectron.,* vol. 240, p. 115603, 2023

[5] D. Jeong and D. Kim, "Super - resolution fluorescence microscopy - based single - molecule spectroscopy," *Bull. Korean Chem. Soc.,* vol. 43, no. 3, pp. 316-327, 2022

[6] M. J. Rust, M. Bates, and X. Zhuang, "Sub-diffraction-limit imaging by stochastic optical reconstruction microscopy (STORM)," *Nat. Methods,* vol. 3, no. 10, pp. 793-796, 2006

[7] J. Canny, "A computational approach to edge detection," *IEEE Trans. Pattern Anal. Mach. Intell.,* no. 6, pp. 679-698, 1986

[8] D. Kim, T. J. Deerinck, Y. M. Sigal, H. P. Babcock, M. H. Ellisman, and X. Zhuang, "Correlative stochastic optical reconstruction microscopy and electron microscopy," *PloS one,* vol. 10, no. 4, p. e0124581, 2015
